# Mooney face stimuli for visual perception research

**DOI:** 10.1371/journal.pone.0200106

**Published:** 2018-07-06

**Authors:** Caspar M. Schwiedrzik, Lucia Melloni, Aaron Schurger

**Affiliations:** 1 Neural Circuits and Cognition Lab, European Neuroscience Institute, Göttingen, Germany; 2 University Medical Center Goettingen, Göttingen, Germany; 3 Department of Neurosurgery, Columbia University, New York, New York, United States of America; 4 Neuroscience Department, Max Planck Institute for Empirical Aesthetics, Frankfurt am Main, Germany; 5 INSERM, Cognitive Neuroimaging Unit, Gif sur Yvette, France; 6 Commissariat à l’Energie Atomique, Direction des Sciences du Vivant, I2BM, NeuroSpin center, Gif sur Yvette, France; Universidad Torcuato Di Tella, ARGENTINA

## Abstract

In 1957, Craig Mooney published a set of human face stimuli to study perceptual closure: the formation of a coherent percept on the basis of minimal visual information. Images of this type, now known as “Mooney faces”, are widely used in cognitive psychology and neuroscience because they offer a means of inducing variable perception with constant visuo-spatial characteristics (they are often not perceived as faces if viewed upside down). Mooney’s original set of 40 stimuli has been employed in several studies. However, it is often necessary to use a much larger stimulus set. We created a new set of over 500 Mooney faces and tested them on a cohort of human observers. We present the results of our tests here, and make the stimuli freely available via the internet. Our test results can be used to select subsets of the stimuli that are most suited for a given experimental purpose.

## Introduction

**Fig 1:**
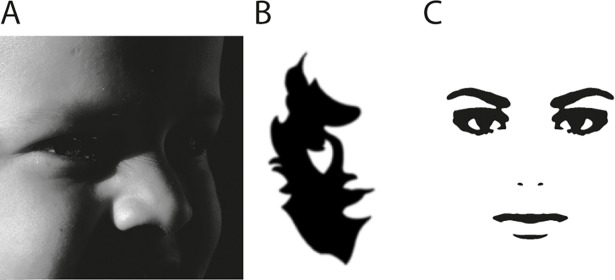
Face and Mooney face stimuli. **(A)** This greyscale image is easily perceived as a face although most visual information is covered by shadows. **(B)** A typical “Mooney” face. **(C)** An extremely easy “Mooney” face, devoid of cast shadows.

One of the hallmarks of vision is the ability to recognize objects on the basis of very little information. For example, a face that has only barely emerged from behind a shadow can often be immediately recognized, even though only a few patches of dark and light are available on the retina (Fig 1A). Missing detail is inferred and the object as a whole (the Gestalt) is perceived based only on a few visual “hints”. This process of visual completion based on prior perceptual knowledge is referred to as “perceptual closure”. In order to study visuo-perceptual closure in children [1], in the 1950’s cognitive psychologist Craig Mooney created a set of 40 human face stimuli, each defined by a few smooth rounded patches of black and white (Fig 1B). Such stimuli can take a few seconds to recognize at first [2], but once the face is perceived it is difficult for a normally-sighted human observer not to see the face from that moment onward [3]. When such a stimulus is presented upside-down it is often not recognized as a face, even if it is immediately recognized as a face when presented upright [4], revealing a hallmark of holistic face processing.

Images of this type, now commonly referred to as “Mooney faces”, have become widely used in cognitive psychology and neuroscience experiments because they offer a means of inducing variable perception with constant visuo-spatial characteristics in humans [5–8] and other animals [9–12]. Non-face objects, such as fruits, houses, and tools, can also be rendered in this way [11, 13–15]. The stimuli are created by first blurring a grayscale image, and then increasing the contrast to 100%, so that only patches of black and white remain. For faces, it is important that the original photograph be taken from an oblique angle and in the presence of shadows, otherwise the resulting stimulus can be trivially easy to recognize (Fig 1C).

Mooney’s original set of 40 stimuli have been used in many studies to investigate closure and face perception, and deficits therein in neurological patients [16, 17], as well as in psychiatric and neurodevelopmental disorders [18–21]. However, it is long known that the small set of Mooney’s original stimuli is quickly overlearned [22], preventing, e.g., longitudinal experiments. Furthermore, for many purposes it is necessary to employ a much larger set of stimuli, e.g., in psychophysical experiments were many trials need to be collected. In addition, it may be important to work with sets of stimuli that are approximately equated in terms of how readily they are recognized when presented upright and when presented inverted. In the past, researchers have resorted to creating their own stimuli [23–25], but this hampers comparisons between studies. Therefore, we created a large set of more than 500 Mooney face stimuli and tested them in healthy human observers using a face detection task. Each stimulus was presented twice, once upright and once inverted, and the subject was asked to indicate, on each trial, whether or not s/he had perceived a face. Scrambled versions of some of the images were included as explicit non-face control stimuli; half of the subjects were shown 50 scrambled images, and the other half 100 scrambled images. An infrared eye tracker was used to control for eye movements. We recorded subject’s responses and reaction times and subjected these and the images themselves to a battery of statistical tests, including a comparison of our stimuli to Craig Mooney’s original set of images. Our stimuli resemble Mooney’s originals in terms of overall difficulty and induce the typical face inversion effect when presented upside-down. They thus lend themselves well to the experimental study of perceptual closure and face perception. The full set of stimuli and the results of our behavioral tests, indexed by stimulus name, are freely available via the internet at https://doi.org/10.6084/m9.figshare.5783037 [26].

## Materials and methods

### Subjects

A total of 20 subjects participated in this study (11 female, mean age 25.5 yrs, range 16–63 yrs, 15 right-handed, as assessed with the Edinburgh Inventory [27]). Subjects were pseudo-randomly assigned to two groups that were shown different numbers of catch trials (50 vs. 100 scrambled faces). One subject of each group was later excluded from analyses because they did not comply with task instructions (final *n* = 18). Because previous studies reported sex-differences in the perception of Mooney faces [28, 29], we made sure that the female:male ratio was the same in both groups. All subjects had normal or corrected-to-normal vision, reported no history of neurological or psychiatric disease, and gave written informed consent before participation. Subjects received monetary compensation for their participation. All procedures were approved by The University Committee on Activities Involving Human Subjects at Columbia University.

### Stimuli

Stimuli were created from images taken from various sources on the internet. We primarily chose images that were taken from an oblique angle and had visible shadows, preferring “artistic” images over mugshots. They were scaled to 160×230 px size, converted to grey scale, smoothed with a 2 px Gaussian kernel, and then binarized at a threshold in Photoshop (Adobe Systems Inc.). Since there is no established threshold to create Mooney faces, the threshold was set by hand based on the subjective impression of the authors. To create scrambled versions of the images, contiguous regions in the images were selected and manually moved around to create distortions without creating sharp boundaries that are not present in the undistorted Mooney face. Subsequently, in pilot experiments, subjects were asked to identify scrambled images in which they still recognized faces. This process was iterated until the number of ‘face’ responses for scrambled images was minimized and subjects agreed in more than 85% of the images that there was ‘no face’.

### Procedures and task

For all experiments, stimuli were presented for 100 ms against a black background on a LCD computer screen (resolution 1600×1200 px, refresh rate 60 Hz), located at 74 cm distance. Stimulus dimensions (7×10 dva) matched previous studies using Mooney faces [18]. Subjects were instructed that they would be shown highly degraded pictures of faces and pictures not containing faces, and that they would have to decide on each trial whether the picture contained a face or not. Subjects used the index finger of their dominant hand to press ‘G’ on a standard computer keyboard for ‘face’, and their middle finger of the same hand to press ‘H’ for ‘no face’. Stimuli were presented in five blocks of 226 or 236 trials, respectively, depending on the number of catch trials (50 vs. 100). The number of upright/inverted/scrambled images was balanced across blocks. A red fixation dot was continuously present at the center of the screen. Stimulus display and response collection were controlled using Presentation software (Neurobehavioral Systems).

### Eye tracking

We used binocular video-based eye tracking at a sampling rate of 500 Hz (Eyelink 1000, SR Research) to assess fixation stability during the experiment in 14 of the subjects and to verify that subjects were looking at the stimuli during the task. A standard 9-point calibration routine was run at the beginning of the experiment as well as after each break.

### Data analyses

Data were analyzed in Matlab (The Mathworks) and SPSS (IBM Corp.). To assess face detection sensitivity, we calculated *d’* as a bias-free measure of face detection accuracy with the loglinear correction to avoid infinite *z*-scores [30]. For the analyses of reaction times, we excluded trials with reaction times shorter than 150 and longer than 2000 ms. The choice of cutoff did not affect the overall pattern of results. To assess inter-rater reliability and internal consistency, we calculated Fleiss’ Kappa [31] and Cronbach’s Alpha [32], respectively. Fixation stability was determined in a 2.5×2.5 dva window around the fixation dot for the time period from -150 to 150 ms around stimulus onset and averaged across both eyes.

## Results

Subjects (*n* = 18) showed generally high Mooney face detection sensitivity, with an average *d’* of 1.19 (SD 0.30) across tasks (upright vs. inverted, upright vs. scrambled, inverted vs. scrambled) and subjects. A mixed repeated measures analysis of variance (rmANOVA) showed that performance did not depend upon how many scrambled images were shown (main effect group *F*(1,16) = 0.06, *p* = 0.79; task×group interaction *F*(2,32) = 0.06, *p* = 0.86, Greenhouse-Geisser corrected); hence, we report aggregate results across all participants. Subjects exhibited the highest face detection sensitivity when we compared behavior for upright faces versus scrambled faces (mean *d’* 1.78, SD 0.45), followed by upright versus inverted faces (mean *d’* 0.94, SD 0.26), followed by inverted versus scrambled faces (mean *d’* 0.83, SD 0.45).

**Fig 2:**
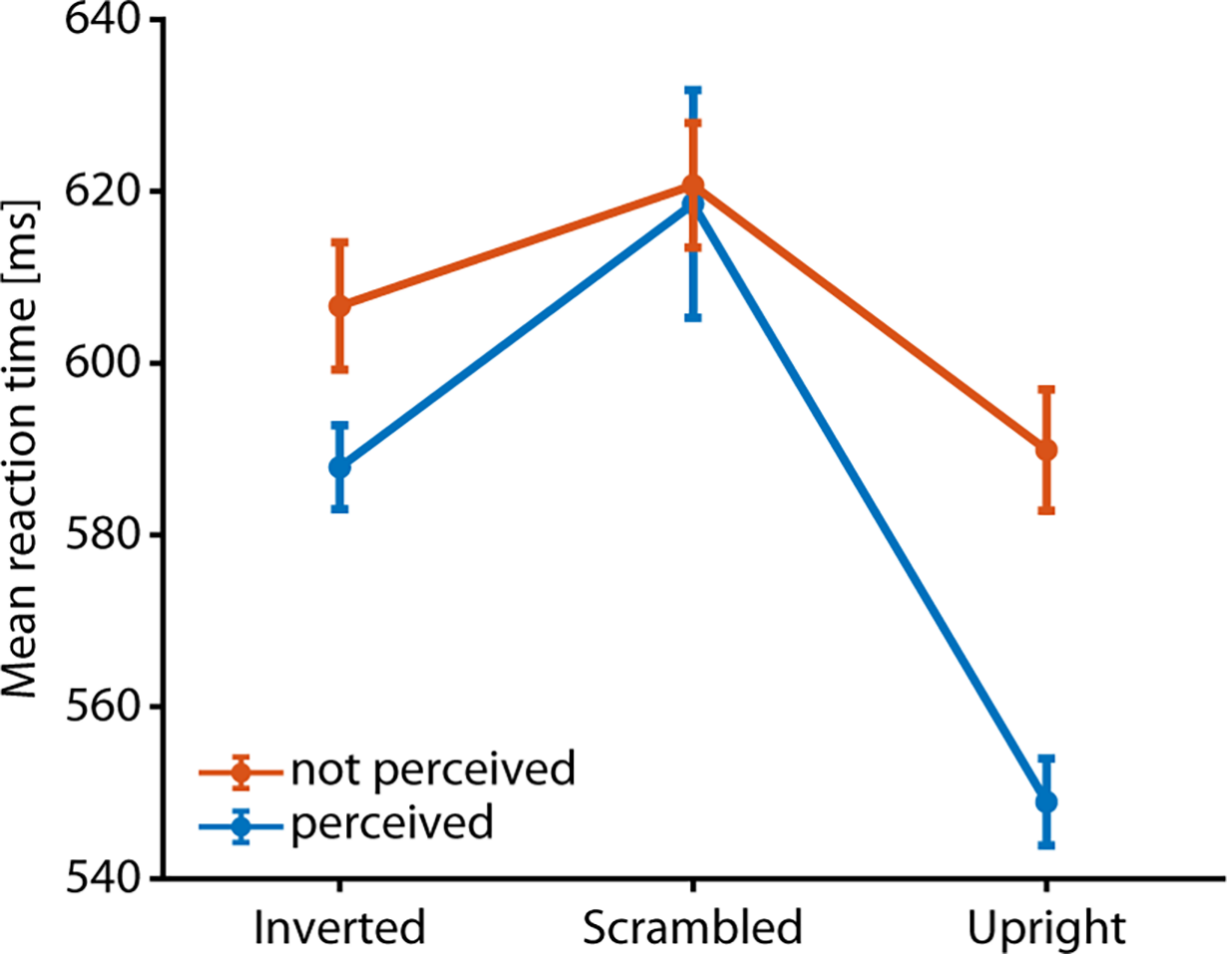
Reaction times. Subjects were faster when they perceived a face then when they did not, both in the upright and in the inverted condition (mean difference 29.8 ms, *T*(17) = 3.86, *p* = 0.001). They also showed a typical face inversion effect, taking longer to perceive inverted than upright Mooney faces (mean difference 38.9 ms, *T*(17) = 7.54, *p*<0.001). No reaction time differences between perceived and non-perceived scrambled images were observed (mean difference 2.1 ms, *T*(17) = 0.11, *p* = 0.906). Error bars reflect the standard error of the mean, corrected for between-subject variability [34, 35].

It took subjects approximately 550 ms (SD 81.5 ms) on average to identify upright Mooney faces (Fig 2), which is slow compared to published reaction times of ~100 ms in speeded face detection with undegraded stimuli [33]. Subjects were generally fastest when they perceived a Mooney face, whether it was upright or not, compared to when they did not perceive a face (mean difference 29.8 ms, *T*(17) = 3.86, *p* = 0.001); the fastest reaction times were observed for upright faces perceived as such (all *p*<0.0013, Bonferroni corrected). We also observed a typical face inversion effect, where subjects took longer to perceive inverted than upright Mooney faces (mean difference 38.9 ms, *T*(17) = 7.54, *p*<0.001). Across stimuli, 16 of 18 subjects showed individually significant inversion effects (*p*<0.05, Wilcoxon signed rank test). For scrambled images, reaction times were very slow (mean 619.6 ms, SD 73.5 ms) and did not differ between trials where subjects reported seeing a face and trials where subjects did not report seeing a face (mean difference 2.1 ms, *T*(17) = 0.11, *p* = 0.906).

Fixation stability during stimulus presentation was generally high (all medians >92%) and did not differ between conditions (no significant main effects or interactions for the factors stimulus (upright, inverted, scrambled) or percept (face, no face), rmANOVA, all *p*>0.7, Greenhouse-Geisser corrected), as expected [36]. Thus, differences in fixation stability did not systematically drive ‘face’ vs. ‘no face’ responses.

Overall, our behavioral results show a typical pattern of performance with Mooney faces where face detection is comparatively slow but accurate, and face inversion further slows but does not fully eliminate face perception.

### Item analyses

**Fig 3:**
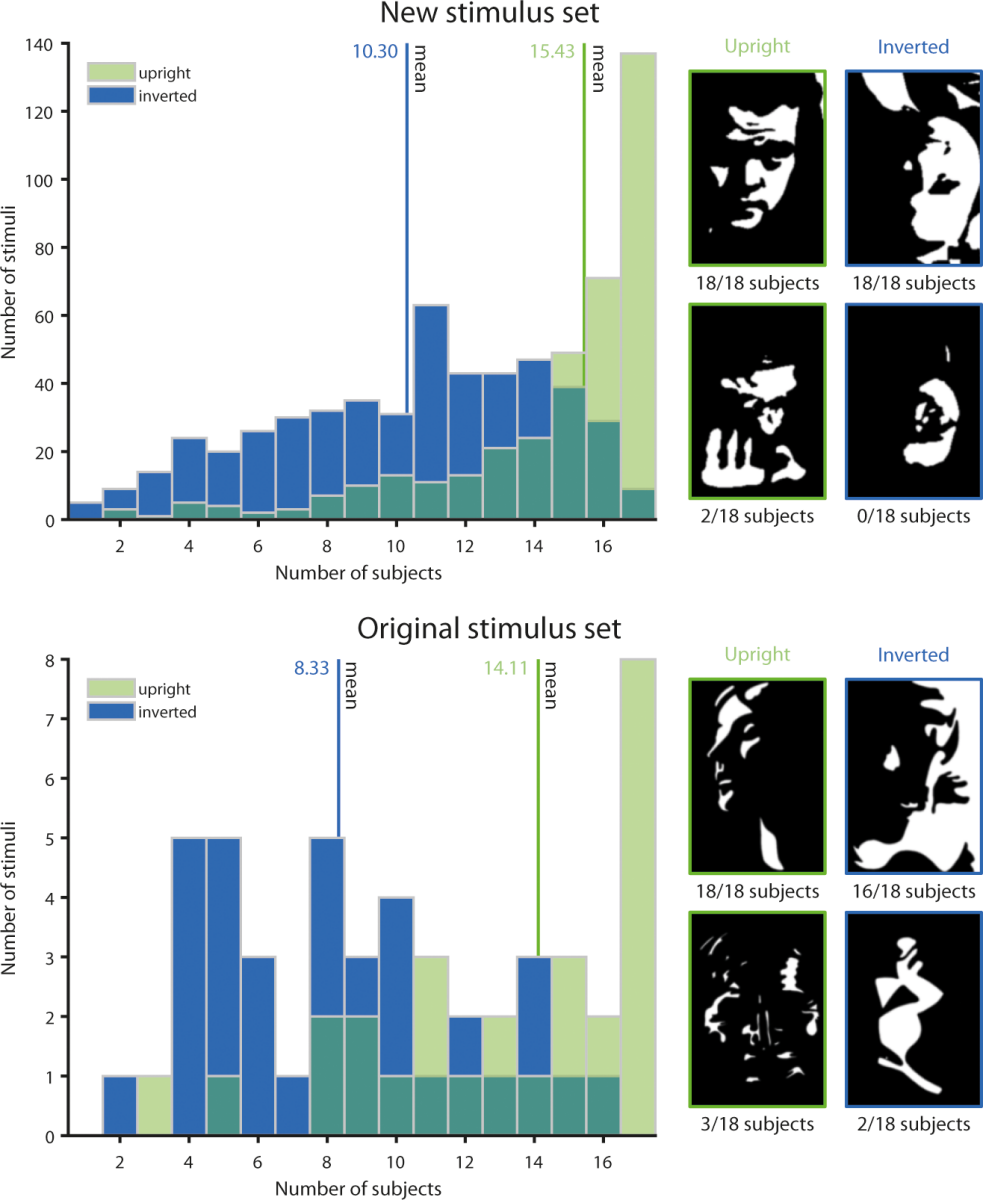
Frequency of stimuli perceived as face, upright or inverted. Most faces were correctly identified as faces by the majority of subjects when presented upright (green) both in our and in the original Mooney face set, but both stimulus sets also contain difficult stimuli that are only perceived as faces by a few subjects. Face inversion (blue) markedly reduced the number of ‘face’ responses. On the right are examples of easy and difficult Mooney faces from the upright and the inverted conditions, respectively. Original Mooney faces reprinted from [1] under a CC BY license, with permission from the Canadian Psychological Association Inc., original copyright 1957.

More than 90% of our new Mooney face stimuli were correctly recognized as a face by at least half of the subjects when presented upright, and all of the new upright Mooney faces were correctly identified as a face by at least two subjects (Fig 3). The most difficult new Mooney faces were recognized by only 2 of the 18 subjects. Overall, successful face recognition ranged from 11 to 100% of subjects per stimulus. For comparison, in the original set of Mooney faces, 86% of upright stimuli were recognized as a face by at least half of the subjects. The most difficult original Mooney face was recognized by 3 of the 18 subjects. The distributions of recognition per item did not differ significantly between ours and Craig Mooney’s original face images (Kolmogorov-Smirnov test, *D* = 0.18, *p* = 0.18). Internal consistency of our upright faces, i.e., the degree of correlation between individual images in terms of whether or not they were perceived as faces, was high (Cronbach’s *α* = 0.97), suggesting that they constitute a comparatively homogeneous pool of stimuli. In contrast, inter-rater reliability, i.e., the degree of agreement in recognizing an upright face as a face across subjects, was only ‘fair’ [37], albeit significant at *κ* = 0.21 (SE 0.003, *Z* = 59.2, *p*<0.0001) for our stimulus set, compared to *κ* = 0.26 (SE 0.013, *Z* = 19.2, *p*<0.0001) for the original stimulus set. This likely reflects inter-individual variability in face recognition and/or perceptual closure capabilities.

When presented upside-down, only 67% of our new stimuli were recognized as faces by at least half of the subjects, ranging from 0 to 100% of subjects per stimulus (Fig 3). In the original Mooney face set, 44% of inverted images were recognized as faces, ranging from 11 to 89% of subjects per stimulus. Again, internal consistency of our stimulus set was high (Cronbach’s *α* = 0.98), while inter-rater reliability was only ‘slight’ [37], both for our (*κ* = 0.15, SE 0.003, *Z* = 41.8, *p*<0.0001) and for the original stimulus set (*κ* = 0.11, SE 0.013, *Z* = 8.53, *p*<0.0001).

**Fig 4:**
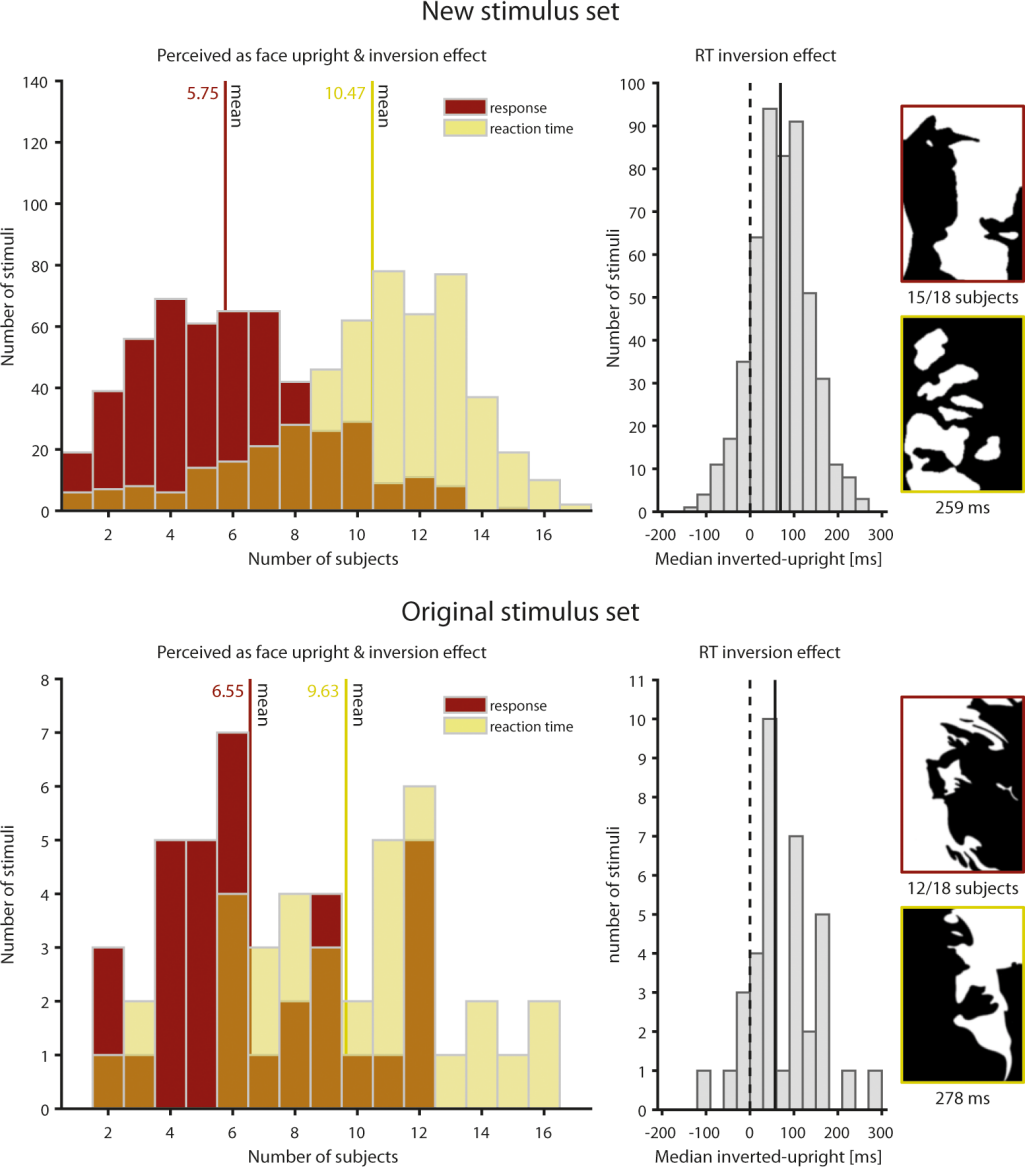
Inversion effects. Inversion effects were evident both when considering whether an inverted face was recognized as a face (red) and when considering reaction times (yellow), i.e., longer reaction times to inverted than to upright faces. The latter type of inversion effect was much more frequent than the former. Overall, inversion effects of reaction times showed a median effect of 66 ms in our new stimulus set, and 79 ms in the original Mooney stimulus set (black solid lines). The right shows the stimuli with the most reliable/largest inversion effects for face recognition and reaction times, respectively. Original Mooney faces reprinted from [1] under a CC BY license, with permission from the Canadian Psychological Association Inc., original copyright 1957.

Mooney faces are often used to investigate face inversion effects. Here, one can consider either face recognition or reaction times to determine whether inversion effects are present (Fig 4). Interestingly, the two types of inversion effects are only weakly correlated (number of subjects recognizing inverted Mooney face vs. median reaction time difference, *r* = 0.09, *p* = 0.03, Spearman rank correlation). When using face recognition as a criterion, 84 images were both recognized as a face when presented upright and not recognized as a face when inverted by at least half of the subjects in our new stimulus set, compared to 11 images in the original stimulus set. Inversion effects in reaction times were much more common, with an overall median of 69 ms (Fig 4). The largest per-image median inversion effect we observed was 259 ms in our stimulus set, and 278 ms in the original Mooney stimulus set. 396 of our stimuli were both recognized as a face when presented upright and showed an inversion effect in reaction times in at least half of the subjects, while this was the case for 22 images in the original stimulus set. In both stimulus sets, faces that were more easily recognized as faces when presented upright were also more likely to be recognized as such when presented upside-down (ours: *r* = 0.67, *p*<0.001; original: *r* = 0.61, *p*<0.001), and induced larger reaction time inversion effects (ours: *r* = 0.30, *p*<0.001; original: *r* = 0.42, *p* = 0.01).

## Discussion

We present a new, large set of Mooney face stimuli that cover a range of difficulty levels and induce typical inversion effects in face perception. The behavior we observe for our new stimuli generally resembles the behavior observed with the original but much smaller stimulus set developed by Craig Mooney in the 1950’s. Together, this suggests that vision researchers interested in face perception and/or perceptual closure can use our freely available stimulus set in situations where large numbers of stimuli are needed, e.g., to prevent learning effects, and/or when stimuli need to be pre-selected to reliably induce certain behavioral effects, e.g., inversion effects in reaction times. Furthermore, our data can provide a reference against which future studies can be compared, thus contributing to reproducibility.

In the past, many studies have relied on the original set of Mooney faces, which are also sorted by difficulty. However, in contrast to the face recognition task we used here, the original task was non-speeded sorting of paper cards into age and gender categories [1]. Stimulus difficulty is correlated between the original task and the more common face recognition task we used (*r* = 0.52, *p* = 0.001), similar to what has been observed for a spatial 3AFC task in a new online version of the Mooney test (*r* = 0.56) [29]. Given the rather weak correlation, it seems advisable to determine stimulus difficulty anew for each new task. However, the face recognition task we used here is likely to tap into more basic perceptual processes than age and gender categorization and thus constitutes a good starting point. We also find that face inversion has two partially distinct effects: slowing of reaction times, and attenuation of recognition abilities. While the former effect occurs for many Mooney face stimuli, including the original ones, the latter is much less frequent (Fig 4), which cautions against the untested use of inverted Mooney faces as non-face stimuli. An alternative is to use scrambled Mooney images which similarly preserve low-level stimulus features, as we did here.

Mooney faces have proven a highly effective stimulus for the investigation of face perception and perceptual closure abilities in development [38–40], disease [16–21], across cultures [41] and species [9–12], from basic vision [42] to aesthetic experience [43], as well as in computer vision [44]. Our new stimulus set, benchmarked against human observers, will serve as a valuable resource for future research.
